# Translucence perception is not dependent on cortical areas critical for processing colour or texture^☆^

**DOI:** 10.1016/j.neuropsychologia.2017.11.009

**Published:** 2019-05

**Authors:** A.C. Chadwick, C.A. Heywood, H.E. Smithson, R.W. Kentridge

**Affiliations:** a Department of Psychology, University of Durham, United Kingdom; b Department of Experimental Psychology, University of Oxford, United Kingdom

**Keywords:** Vision, Translucence, Perception, Cerebral cortex, Cerebral achromatopsia

## Abstract

Translucence is an important property of natural materials, and human observers are adept at perceiving changes in translucence. Perceptions of different material properties appear to arise from different cortical regions, and it is therefore plausible that the perception of translucence is dependent on specialised regions, separate from those important for colour and texture processing. To test for anatomical *independence* between areas necessary for colour, texture and translucence perception we assessed translucency perception in a cortically colour blind observer, who performs at chance on tasks of colour and texture discrimination. Firstly, in order to establish that MS has shown no significant recovery, we assessed his colour perception performance on the Farnsworth-Munsell 100 Hue Test. Secondly, we tested him with two translucence ranking tasks. In one task, stimuli were images of glasses of tea varying in tea strength. In the other, stimuli were glasses of tea varying only in milkiness. MS was able to systematically rank both strength and milkiness, although less consistently than controls, and for tea strength his rankings were in the opposite order. An additional group of controls tested with greyscale versions of the images succeeded at the tasks, albeit slightly less consistently on the milkiness task, showing that the performance of normal observers cannot be transformed into the performance of MS simply by removing colour information from the stimuli. The systematic performance of MS suggests that some aspects of translucence perception do not depend on regions critical for colour and texture processing.

## Highlights

• Cortically colour blind MS systematically ranks stimuli varying in translucence.
• These rankings are less consistent than controls, and some in the opposite order.
• Aspects of translucence perception do not depend on colour processing regions.

## 1 Introduction

One of the key insights that prompted the first studies of blindsight (Pöppel et al., 1973) was that simple visually controlled functions such as the pupillary light reflex and optokinetic nystagmus remain intact in patients with cortical lesions that render them subjectively blind. Weiskrantz and his colleagues demonstrated that these simple functions were the tip of an iceberg of visual faculties that operate independently of visual consciousness (e.g. Weiskrantz, 1997). These findings demonstrate the frailty of introspection – neurological conditions such as blindsight show that our visual systems do not necessarily operate as we feel they do. Functions within vision dissociate in unexpected ways. In cerebral achromatopsia (cortical colour blindness) dissociations have been found between perception of colour and perception of shape and motion defined by solely colour. That is, if shown a stimulus in which a figure differs only in colour from its background, a patient who cannot see or discriminate the colours of the figure and background nevertheless effortlessly sees the figure (quite consciously, Heywood et al., 1998a) and perceives motion defined only by movements of colours (Heywood et al., 1998b). In this paper we test a patient with cerebral achromatopsia in order to investigate whether the perception of translucence dissociates from other aspects of material perception that are lost in cerebral achromatopsia. The implication of any such dissociation would be that cortical areas involved in translucence perception are anatomically distinct from those involved in colour or texture perception (both of which are lost in cerebral achromatopsia, Cavina-Pratesi et al., 2010a, Cavina-Pratesi et al., 2010b).

### 1.1 What is translucence?

**Fig. 1:**
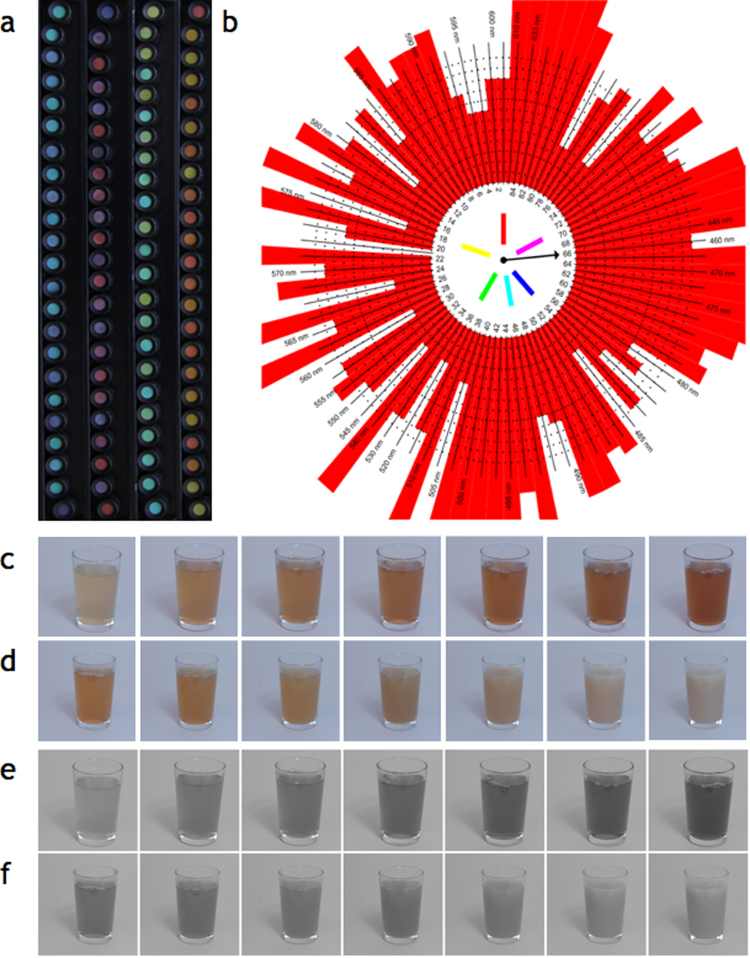
a) The Farnsworth-Munsell 100 Hue Test colour chips as ordered by MS. b) MS's performance on the Farnsworth-Munsell Test. c) and d) show the colour stimuli: images of glasses of real milky tea, with c) varying in tea concentration only and d) varying in milk concentration only. e) and f) show the greyscale versions of the stimuli, where e) varies in tea concentration and f) varies in milk concentration.

Visual identification of materials requires the ability to visually discriminate a range of properties such as colour and texture. Many natural materials not only reflect light from their surfaces but are also translucent. Translucence is an optical characteristic caused by light scattering below the surface of an object, within the material. The two most important physical parameters that determine how light is transported within a material are the scattering and absorption coefficients, which essentially capture the rate at which light spreads and is attenuated as it travels through the material. These parameters differ between commonly occurring substances, and determine their appearance. An ability to discriminate them could provide behaviourally relevant information about the material world. These properties vary depending on the ripeness of fruit, the condition of skin, the freshness of meat, and the muddiness of water. Translucence is not directly associated with any simple low-level cues like lightness or colour saturation. Fig. 1 shows photographs of two sets of stimuli varying in translucence. Panels c) and d) are colour photographs; panels e) and f) are greyscale versions of the same photographs. There are individual stimuli in panels c) and d) and in panels e) and f) that have the same lightness, but differ greatly in their perceived translucence properties.

### 1.2 Our ability to perceive translucence

Making judgements of the strength and purity of mixtures of translucent materials (e.g. how strong tea is and how milky) is not simply a matter of identifying a material as being translucent, but also of estimating its scattering and absorption parameters. Judgements of strength and purity in translucence are perceptually separable (Chadwick et al., under submission). However, such judgements do not map directly onto the underlying physical parameters of scatter and absorption. In addition, translucence is conceptually distinct from material properties such as colour, gloss and texture. Perceived translucence therefore appears to depend on pseudocues (i.e. cues that the visual system extracts from the scene, which act as heuristics rather than deterministic cues to the physical properties being perceived - Chadwick and Kentridge, 2015), which are indirectly driven by changes in the underlying physical parameters, but that can also encapsulate complex interactions of light transport within the material. Factors affecting perceived translucence include the amount of light absorption (darkness), the direction of illumination, the shape and size of the object, and colour (Fleming and Jensen, 2004; Fleming and Bülthoff, 2005; Fleming, 2014). Human observers are capable of identifying materials within fractions of a second (Sharan et al., 2009), and can readily discriminate visually between very similar translucent materials such as skin or fruit and their waxwork replicas, despite the subtlety of such differences. Many of these fine differences are thought to be due to the differing properties of translucent layers at the surface of materials. Recent research has focused on potential cues for making judgements of translucence: the relationship between shading patterns and specular highlights across space has been suggested as an important cue for perceptual translucency (Motoyoshi, 2010), and, consistent with this approach, it does appear that perceptual translucency is dependent on local image features within specific image regions, rather than on global luminance statistics (Nagai et al., 2013). There has, however, been little focus on the cortical areas that may be involved in human translucence perception. As we appear to be so proficient at detecting differences in translucence (Jensen et al., 2001, Vasseleu, in Cubitt et al., 2015, p. 163–178, and Murakoshi et al., 2013), and translucence seems to be associated with important properties of natural materials such as the health of skin or ripeness of fruit (Hetherington et al., 1990, Fleming et al., 2004, Fleming and Bülthoff, 2005), it is plausible to ask whether there is a region of cerebral cortex specialised for processing translucence.

### 1.3 Cortical responses to material properties

It is already clear that the properties that contribute to material identification are not all processed at the same neural locus. Cant and Goodale (2007) first demonstrated that attending to material properties or to shapes of objects activates distinct regions of cortex. Cavina-Pratesi et al., 2010a, Cavina-Pratesi et al., 2010b went on to show that texture and colour discrimination rely on distinct areas of cerebral cortex – posterior collateral sulcus vs. anterior collateral sulcus and lingual gyrus - and in between the two there is a region activated by both colour and texture. Kentridge et al. (2012) tested neurological patient MS, who lacked the areas implicated in processing texture and colour and found that he could, nevertheless, discriminate glossiness independently of variation in lightness or texture. It was inferred that perceived glossiness must therefore be processed by an area of cortex distinct from areas responsible for colour and texture processes that MS had lost. Nishio et al. (2012) also found evidence in the monkey brain to support that idea that the perception of gloss may not depend on the same areas as the perception of colour and visual texture.

### 1.4 Experimental approach

We used the same logic as we applied in Kentridge et al. (2012) to test whether translucence perception was dependent on a neurally distinct locus from colour and texture perception. If patient MS, who has lost these colour and texture areas, can, nevertheless, discriminate differences in translucence this suggests that translucence perception must depend on regions distinct from those mediating colour and texture perception.

To test MS's ability to perceive translucence, we asked him to rank translucent stimuli that varied in their absorption and scattering of light. To be clear, we were not directly testing the role of *colour* in translucence perception. Our stimuli were photographs of glasses of milky tea, in which the concentration of tea primarily affects the absorption of light and the concentration of milk primarily affects the scattering of light. We have previously compared people's perception of translucence in real and computer-generated stimuli (Chadwick et al., under submission) and found that people respond differently to computer-rendered images in which scattering and absorption from milk and tea do not interact naturally. In particular, we showed that the two dimensions of tea strength and milkiness were better separated in real tea. We therefore used photographs of real tea stimuli in the current study (details of creation can be found in the Methods and Supplementary materials).

The case would be further strengthened by showing that MS was not using low-level visual properties like brightness statistics to make these discriminations. In addition to MS, we tested a small number of control participants (one age-matched, and two non-matched) with the same, coloured versions of the stimuli, and a further three control participants with monochromatic versions of the stimuli to test the extent to which the performance patterns of MS might be explained by his lack of colour vision. For both MS and controls, we compared performance to computer simulations based on low-level visual properties.

## 2 Methods

### 2.1 MS's case history

Following brain damage as a result of suspected viral encephalitis when aged 22 in 1970, MS, aged 67 at the time of testing, developed dense achromatopsia (colour blindness of cortical origin) accompanied by a left hemianopia (with macular sparing) prosopagnosia and visual object agnosia (Heywood and Kentridge, 2003, Kentridge et al., 2004); however, he has intact Snellen acuity (Mollon et al., 1980). He performs at chance on tasks of colour and texture discrimination, but above chance on tasks of glossiness discrimination (Cavina-Pratesi et al., 2010b; Kentridge et al., 2012). Neuroimaging indicated that MS lacks the regions normally activated by texture and colour in healthy observers (Cavina-Pratesi et al., 2010a, Cavina-Pratesi et al., 2010b). Structural MRI (Heywood et al., 1991) revealed extensive bilateral lesions to ventromedial occipito-temporal cortex, damage to the 2nd, 3rd, 4th, and 5th temporal gyri in the right hemisphere, and damage to the right temporal pole. Right striate cortex was completely destroyed, which accounted for the left field hemianopia. In the left hemisphere, there was damage to the left temporal lobe, confined to the left temporal pole, 4th temporal gyrus, and hippocampal gyrus. There was also bilateral ventral-occipital damage to the lingual and fusiform gyri.

### 2.2 MS's colour vision

We established that MS's achromatopsia remained unchanged by asking him to complete the Farnsworth-Munsell 100 Hue Test, a colour vision task which determines the ability of an observer to sort and order sets of coloured chips. It is used to identify types of colour blindness. MS was asked to arrange a number of equiluminant chips in chromatic order. There are four boxes of chips, and for each there are ‘anchor’ chips at either end, between which the participant must arrange the rest of the chips for that box. In an early test, MS had scored 1245 (Mollon et al., 1980) - Fig. 1a shows the hue chips as ordered by MS, seemingly placed at random within each box, and Fig. 1b illustrates the test scoring. The graph of a participant with normal colour vision would show a small ring of red around the centre; a graph for a dichromat would show spikes in the direction of their colour blindness (at red-green, or blue-yellow). MS's total error score was 1268, which confirms that MS has not recovered any colour vision in over three decades. A score would be expected to be in the range of 170–195 for a normal age-matched observer (Kinnear and Sahraie, 2002) – the worst 5% of performances in the normal population score between 80 and 195, depending on age. Previous performances of achromatopsic observers result in a mean score of 582 (Bouvier and Engel, 2006). However, MS's score reflects random responding, which would correspond to a score of approximately 1200 (Victor, 1988).

### 2.3 Stimuli: photographs of tea varying in strength and in milkiness

To test participants’ abilities to perceive translucence, we asked them to rank photographs of milky tea that varied in their absorption and scattering of light. One stimulus set was produced with a fixed level of milkiness and increasing strength, and a second stimulus set was produced with a fixed level of strength and increasing milkiness. We asked observers to rank order the first set on the basis of strength of tea (primarily dependent on absorption), and the second set on the basis of milkiness (primarily dependent on light scattering). Each set had seven levels of concentration of black tea or milk respectively (Fig. 1c and d), chosen to produce approximately equally discriminable steps across the range. Further details of stimulus creation are included in Supplementary materials.

### 2.4 Procedure: ranking of tea strength and milkiness

On each trial, the observer was shown three stimuli (each subtending nine degrees of visual angle square) arranged vertically in the centre of the screen (MS's hemianopia makes comparison of vertically arranged stimuli easier than horizontally arranged ones). Judgements of tea strength and of milkiness were obtained in separate sessions. On each trial, three stimuli were selected from the set of seven, and the observer was asked to rank them, either from the lowest to the highest tea strength, or the lowest to highest milk concentration. For each task, we used all unique combinations of three (k) stimuli selected from seven (n) given by the binomial coefficient, n!/((n–k)! k!), to yield 35 trials.

Initially, we determined whether MS understood what it meant for a liquid to look more or less absorbing, or more or less cloudy. He described the liquids as either “looking more like beer than water” (indicating a liquid with a stronger concentration of tea), and agreed that some appeared more like milk than water (indicating a liquid with a higher concentration of milk). MS requires additional time to complete trials and fatigues fairly quickly, so completed just one block of trials. In interpreting the results from MS, it is important to remember he has additional cognitive deficits, and so above chance performance even with additional time would remain impressive.

Controls were asked to complete two blocks of trials. Their performance is generally so good that we tested only three controls with colour stimuli (one age-matched, RC; and two non-matched, HR and YB), and tested a further three controls (SK, AC and LN) with greyscale versions of the stimuli).

To make the claim that MS is genuinely discriminating translucence we want to be sure he is not simply responding on the basis of some trivial low-level cue. So, using computer simulation, we test whether any simple image statistics could predict performance. This was not an attempt to find the pseudo cues that people may normally use in making translucence judgements, but rather to characterise the low-level information in our stimuli that might correlate with observers’ judgements.

## 3 Results

### 3.1 Rankings of colour stimuli

**Fig. 2:**
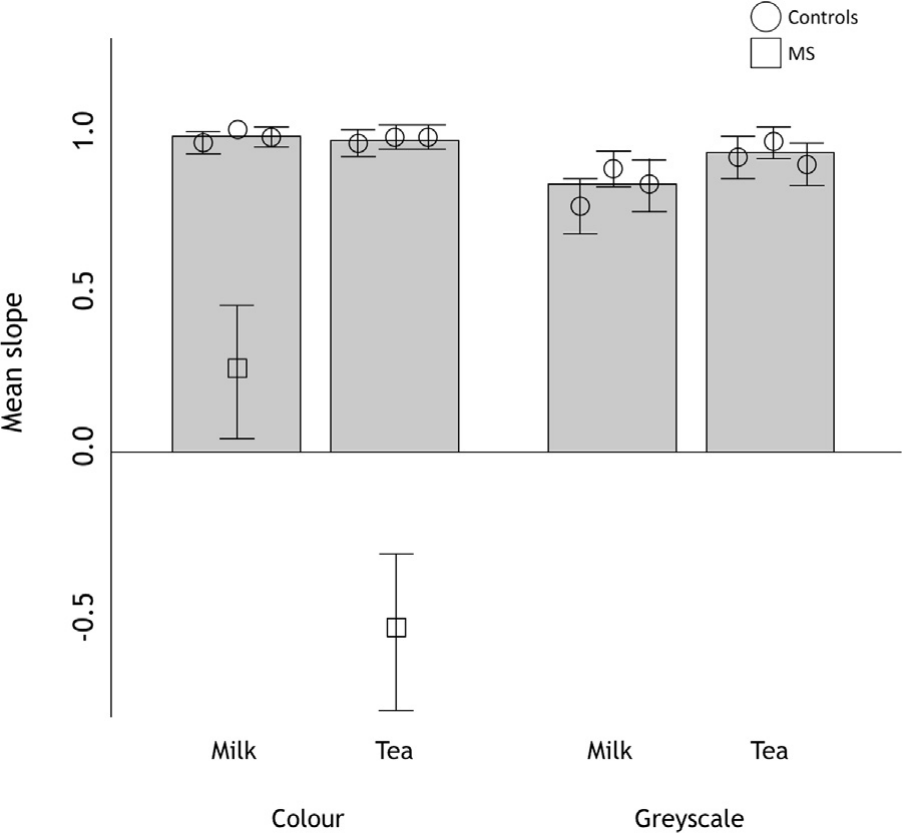
Ranking performance for MS and controls. Mean slopes are plotted as separate symbols for MS and each of the controls for the milk and tea tasks with colour stimuli, and for controls on the greyscale versions of the tasks. Error bars show 95% confidence intervals. Grey bars show mean slopes averaged across controls.

We scored performance by calculating the ‘slope’ of rankings on any trial. Regardless of the absolute values of strength we simply ordered the physical strengths 1, 2, and 3. We then regressed the order of judgements onto these. So, if the reported order, 1, 2, 3, matches the physical order the slope would be 1.0, if the reported order was 3, 2, 1, the slope would be −1.0, if the reported order was 1, 3, 2 the slope would be 0.5 and so on. Slopes are plotted in Fig. 2. For each participant, we then performed a single-sample *t*-test on the slopes of the rankings, where the null hypothesis (if judgements were random) was that the average slope would be zero. Since our task and study-design minimises trial-to-trial correlations, we treat each observation of slope as independent, as required for a t-test. When ranking milk concentration, MS ranked the stimuli significantly non-randomly (differently from 0; m = 0.26, sd = 0.62, t(34) = 2.44, *p* = 0.02) in the correct direction. When ranking tea strength, MS ordered the stimuli significantly non-randomly (t(34) = −5.16, *p* < 0.001) but in the ‘wrong’ direction (m = −0.53, sd = 0.61). All controls ranked the stimuli significantly non-randomly for both tasks, in the correct direction (see Table 1 for *t*-test values). We then tested the difference between the single case mean of MS and the control sample means, to determine whether MS's results were significantly different from those of the age-matched control and the two non-age-matched controls, using single-case methodology for a small sample (Crawford and Garthwaite, 2002). MS's results were significantly different from the controls for both the milkiness task (t(3) = −29.01, *p* < 0.001) and the tea strength task (t(3) = −157.74, *p* < 0.001). The estimated percentage of the normal population falling below MS's score for the milkiness task was 0.059%, and 0.020% for the tea strength task.

**Table 1:** *T*-test values for controls on both the milk and tea tasks.

	t	Df	Sig. (2-tailed)	Mean	Std. Deviation
HR milk	56.802	69	0.000	0.9571	0.14098
HR tea	41.085	69	0.000	0.9643	0.19637
YB milk	*	69	*	1.0000	0.00000
YB tea	45.667	69	0.000	0.9786	0.17928
**RC milk	80.269	69	0.000	0.9786	0.10200
**RC tea	45.667	69	0.000	0.9786	0.17928

Consistent with the t-test results, an assumption-free permutation test, using 10000 simulated slope estimates based on random responding in response to the trials presented, gives p-values < 10^-4^ for observing slopes as extreme as the ones observed, for all participants in all conditions, apart from one: MS’s judgements of milkiness gave a mean observed slope of 0.2571, associated with a p-value of .0142

* This participant was correct on every trial for the milkiness task, and so a *t*-test could not be conducted.

** This participant is age-matched to MS.

### 3.2 Rankings of greyscale stimuli

The decisions made by MS were not random, but they were also far less accurate than those made by the controls. One obvious reason why MS might be so much poorer at ranking tea strength and milkiness is that he has no access to colour information. We therefore tested three additional normal controls on the same two ranking tasks, but using greyscale images (see Fig. 2), to see if absence of colour alone accounts for the performance of MS.

All three control observers were able to rank the greyscale stimuli well, with slopes that were significantly non-random, on both tasks. Performance on the tea task was similar to that of controls tested with colour images (SK: m = 0.91, sd = 0.28, t(69) = 27.13, *p* < 0.001, AC: m = 0.97, sd = 0.24, t(69) = 34.00, *p* < 0.001, LN: m = 0.90, sd = 0.29, t(69) = 25.97, *p* < 0.001) but performance was not as consistent for the milkiness ranking task (SK: m = 0.77, sd = 0.39, t(69) = 16.67, *p* < 0.001, AC: m = 0.89, sd = 0.26, t(69) = 28.75, *p* < 0.001, LN: m = 0.84, sd = 0.26, t(69) = 20.09, *p* < 0.001).

### 3.3 Image statistics

**Fig. 3:**
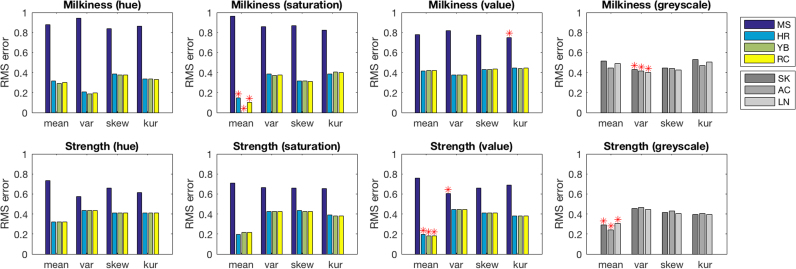
Goodness-of-fit data between real observers and simulated observers using simple image statistics. Plotted values are root-mean-squared (RMS) errors between the trial-by-trial slopes for each observer and for a simulated observer whose decisions are driven by the image statistic under test. The upper panels show data from the milkiness task, and the lower panels show data for the strength task. Each plot summarises measures for different image statistics: hue, saturation and value for the coloured stimuli, and value for the greyscale stimuli. Different groups of bars show mean, variance, skew and kurtosis of the distribution of the relevant statistic from the image. Individual bars show fits for individual participants: MS and three controls (HR, YB, RC) with the coloured stimuli, and three additional controls (SK, AC, LN) for the greyscale stimuli. Asterisks identify the image statistics that produce the best fits, associated with the lowest RMS errors.

If we are to be able to infer anything about MS using a functionally specialised cortical area for translucence perception, distinct from that which he has lost, then we need to rule out the possibility that he may have been ordering translucence stimuli on the basis of some low level visual property. We analysed the images to obtain the common metrics of hue, saturation and value that are used to describe the components of colour. We computed the mean, variance, skewness, and kurtosis of the distributions of hue, saturation and value in the regions of the stimuli occupied by the test liquids. We used the relative magnitudes of a candidate statistic from each image to provide the decision variable for simulated responses in the ranking task. For a set of three stimuli, the simulated decision ranked the images according to the rank order of the statistic chosen. Some metrics are positively correlated with an increase in milkiness or strength (e.g. an increase in strength produces an increase in mean saturation within the image), whereas others are negatively correlated (e.g. an increase in milkiness produces a decrease in mean saturation within the image). For our simulated observers, we assumed the sense of the ranking could be reversed to produce correctly ordered rankings on each task. We then calculated the goodness of fit between these simulated responses and the data obtained from MS and each control participant (see Fig. 3). The upper-bound on MSE values calculated in this way, for a simulated observer that performs perfectly and a real observer who is at chance, or vice versa, is 1. For MS, we reversed the sense of MS's milkiness rankings, since his slopes are consistently negative for this task. Without this step, the goodness-of-fit measures for this task and the candidate metrics would have been poorer.

For the milkiness task, mean saturation provided the best fit to the rankings for all controls (mean squared error, MSE, values for HR, YB, RC = 0.021, 0.000, and 0.011 respectively). For MS, the best fits against simple statistics were very poor. Kurtosis of value provided the best fit, with a large MSE (0.564). For the tea strength task, mean value provided the best explanation for the responses for all controls (MSE values for HR, YB, RC = 0.039, 0.032, 0.032 respectively). Again, for MS, no simple statistic provided a good fit to his responses, with the best fit provided by variance of hue with a large MSE (0.329). Of course MS is extremely unlikely to be relying on hue in any form as a cue as we know MS is unable to discriminate hue. Variance of value provided the next best fit, with a MSE of 0.364.

For controls, the low MSE values for mean saturation and mean value in the milkiness and strength tasks suggest that mean saturation and mean value might have been factors in driving their judgements. However, these image statistics correlate with the physical parameters of milkiness and strength that we asked them to judge. The good fits are therefore not diagnostic of the underlying cues that observers use, since the statistics we have tested are likely to correlate with other image properties that also vary with the physical parameters of tea and milk concentrations. Importantly, though, the high MSE values we calculate for MS suggest that no simple statistic can account for his decisions. As MS's decisions are still, however, far from random, this suggests he must be relying on a more complex analysis of the stimuli in judging translucence properties.

For the data from controls with greyscale stimuli, goodness-of-fit was calculated for simulated performances based on image statistics of value only (see Fig. 3). For the milkiness task, variance of value provided the best fits (MSE = 0.186, 0.171, 0.161 for SK, AC and LN respectively), and for the strength task, mean of value provided the best fits (MSE = 0.086, 0.057, 0.093). For both tasks, however, performance with the greyscale images was less well accounted for by simple statistics than the performance of controls tested with colour images, which suggests that normal observers judging greyscale stimuli may too be basing their decisions on more complex analyses of the stimuli. It appears that depending on the information available, we are flexible in the cues upon which we base our translucence decisions.

## 4 Discussion

MS orders translucent stimuli systematically despite his extensive brain damage. This cannot be accounted for by reliance on low-level image statistics as simulating responses based on these statistics never provided a good fit to MS's pattern of responses. MS's performance is poorer than that of normal observers (who are more or less perfect at the tasks we used), however, MS's performance deficit is not simply due to his inability to access colour information as controls still performed very well even when judging greyscale stimuli.

MS makes systematic rankings of the stimuli for both milkiness and tea strength tasks. Since MS's rankings were significantly different from chance, he must therefore be capable of discriminating between the stimuli. One interesting aspect of the results is that on the tea strength task, MS ranked stimuli consistently, but in the opposite order to controls. It is possible that MS may simply be unable to access the absolute lightness values that may inform his judgements and so, for example, ranked from ‘lightest’ to ‘darkest’ where controls would have ranked in the opposite direction. Although most discussions of cerebral achromatopsia emphasises the deficits in colour vision, it is also the case that MS's perception of greyscale attributes is far from normal (2004). On the milkiness task, MS ranked the stimuli in the same direction as controls, although less consistently. In extensive pilot work, we confirmed that MS had understood the instructions he was given on both tasks.

The behavioural simulations based on image statistics showed that the statistic of best fit to control observers on the milkiness task was mean saturation. For the tea task, the best fit for all control observers was mean value (although a poor fit overall). The statistics of best fit for MS's performance were kurtosis of value and variance of hue respectively (with variance of value giving a similar result). The high residual error in these fits suggest that these low-level image properties are unlikely to be the basis of MS's decisions, and the low fits overall with statistics of hue and saturation are also consistent with MS's inability to perceive colour in other tasks. Is it correct to characterise MS's poor performance is simply due to his inability to access colour information? If so, then we would expect that control observers would also perform similarly poorly without colour information - that is, with greyscale versions of the stimuli. We therefore tested three additional controls with greyscale versions of the tasks.

The performance of observers completing the greyscale task remained good, as can be seen in Fig. 2. Their orderings were perfect on 81.4% of trials. They did report that they found this task harder than ranking colour images, and performance with colour stimuli was indeed better (96.7% perfect orderings). Performance of controls with greyscale images was not qualitatively different from that with colour images in that there is no change in the way they order stimuli in general. Colour information appeared to be beneficial for normal observers in making judgements, especially of milkiness. However, while colour might be a useful cue when making judgements of translucence, the results of the controls with greyscale images show that some cues to translucence perception do not rely on colour information. MS may therefore be able to exploit these cues.

The performance of MS is still poor in comparison to controls’ performances with greyscale stimuli. This may be due to an inability to process all of the non-chromatic cues that controls exploited, or his brain damage may have partially affected neural systems involved in translucence perception. Alternatively, it may just be due to more general factors affecting his memory, attention and behaviour; MS has deficits that make doing all tasks difficult, and so his significantly above chance performance on this task is of great import. Whatever the tissue that MS still has working in the service of translucence perception, it is doing more than simply responding to lightness, hue or saturation or any simple statistical description of their variation. We conclude that there are stimulus attributes processed in specialised cortical regions, which are still intact in MS, and are distinct from colour and texture areas, and that support the perception of translucence.

Following cortical damage, MS shows a dissociation between the perception of colour and texture (which is lost) and the perception of translucence (which is preserved). This study of functional sparing in a neuropsychological case study underlines Larry Weiskrantz's legacy, reminding us not to rely on semantic descriptions (such as cortical blindness, or cortical colour blindness), but to test precisely the features of stimuli whose processing is preserved.
