# Supplementary material for: Translucence perception is not dependent on cortical areas critical for processing colour or texture
Source: Neuropsychologia. 2019 May;128:209–14. doi: 10.1016/j.neuropsychologia.2017.11.009 (PMC6562271; doi:10.1016/j.neuropsychologia.2017.11.009)
Supplement: Supplementary file 1 — Supplementary material [file mmc1.doc]

**Supplementary Materials**

Method of creating the real tea stimuli

Stimuli

To create controlled images of real tea, a ‘master’ tea solution was made with freshly boiled water before adjusting the strength by watering down as required. This volume was kept at a constant temperature to prevent the tannins precipitating and making the volume cloudy, as it was found that there was a just noticeable difference in the spectral composition of the light reflected from a glass of the tea solution detected by a spectroradiometer (Mahy et al. 1994) over a period of fifteen minutes as the liquid cooled. A procedure was developed to create the optimal strength of master solution. The same master solution was used for all stimuli. The total volume of liquid in each glass was 70ml, and the different stimuli were created by varying the amounts of tea solution, water, and milk (semi-skimmed, 1.8% fat).

An initial set of 20 stimuli was produced: 10 varying in milkiness, at the lowest strength of tea, and 10 varying in tea strength at the lowest level of milkiness. After piloting with multiple normal participants, an approximately linear perceptual scale was identified for each parameter within the range of stimuli, consisting of seven levels of milkiness while tea strength was held constant, and seven levels of tea strength while milk concentration was held constant.

Photographing the stimuli

Stimuli were photographed using a calibrated Nikon D80 camera. Glasses of liquid were positioned against a white infinity-curve backdrop, and the scene was lit by an overhead halogen lamp and a single fluorescent desk lamp with daylight spectrum.

Apparatus

Experimental software was written in Matlab. Stimuli were presented on a calibrated NEC 2070SB CRT display (1064x768 pixels with refresh rate of 100Hz), controlled by means of a CRS Visage (Cambridge Research Systems). Observers were seated approximately 100cm in front of the screen in a blacked-out cubicle. Responses were recorded by the experimenter.

Supplementary Material Reference

Mahy, M., Eycken, L., & Oosterlinck, A. (1994). Evaluation of uniform color spaces developed after the adoption of CIELAB and CIELUV. *Color Research & Application, 19*(2), 105-121.
